# Remarkable Temperature Sensitivity of Partially Carbonized Carbon Fibers with Different Microstructures and Compositions

**DOI:** 10.3390/ma14227085

**Published:** 2021-11-22

**Authors:** Zijin Liu, Jun Wang, Chang Li, Cheng Zheng, Bin Zhang

**Affiliations:** 1 School of Materials Science and Engineering, Wuhan University of Technology, Wuhan 430070, China; liuzijin1996@163.com; 2 Institute of Advanced Material Manufacturing Equipment and Technology, Wuhan University of Technology, Wuhan 430070, China; 3 Department of Mechanical Engineering, City & Guilds Building, South Kensington Campus, Imperial College London, London SW7 2AZ, UK; 4 Research Institute of China Shipbuilding Industry Corporation, Wuhan 430200, China; tq029qb@163.com; 5 School of Materials Science and Engineering, Wuhan Textile University, Wuhan 430200, China; whutfrpzhangbin@163.com

**Keywords:** carbon fibers, temperature sensitivity, graphitized-like structure, conductive mechanism

## Abstract

In order to explore effect of structure on the temperature sensitivity of partially carbonized carbon fibers, different heat treatment temperatures (700, 750 and 800 °C) and heat treatment times (3 and 9 min) were used to prepare fibers with different structures. The electrical resistivities were monitored whilst the room temperature was increased from 30 to 100 °C, which was used to characterize the temperature sensitivity. The fibers showed negative temperature coefficients in the temperature range. Infrared spectra, an element analysis, a scanning electron microscope (SEM), Raman spectroscopy and X-ray diffraction measurements were used to study the microstructure of the fibers. Through the analysis, the proportions of the graphite-like structure, graphitization degree and size of the graphite-like structure crystallite influenced the temperature sensitivity. The main electron transfer method used for the fibers was variable-range hopping. This indicated that the fibers had a potential application of preparing thermistors in polymer composites.

## 1. Introduction

Carbon fibers are widely used in the aerospace, automotive and wind energy sectors as well as in marine, construction and biomaterials because of their excellent properties such as a high tensile strength (~7 GPa), a high tensile modulus (~588 GPa), low densities (1.75–2.00 g·cm^−3^), good electrical conductivity (~65 μΩ·cm), inertness and biocompatibility [1,2,3,4,5,6,7,8,9,10,11,12,13]. Common types of carbon fibers include polyacrylonitrile (PAN)-based carbon fibers, pitch-based carbon fibers, cellulose-based carbon fibers, lignin-based carbon fibers and polyethylene (PE)-based carbon fibers [1,14,15,16,17,18,19,20]. For pitch-based carbon fibers, there are two typical procedures for the preparation of the fibers, melt spinning and carbonization [1,3,21]. PAN-based carbon fibers serve as the principal precursor material for carbon fiber production [3]. The preparation of PAN-based carbon fibers is a complicated process that comprises polymerization, stabilization, carbonization and graphitization [22,23,24,25,26,27]. There are many obvious changes that occur in PAN fibers at different stages. Stabilization, cyclization, dehydrogenation and oxidation take place, which can promote the change from linear structures to cyclized structures [3]. In carbonization, the cyclized structures undergo a dehydrogenation reaction and transform into a graphite-like structure. With an increase in the heat treatment temperature, graphite layers are formed by denitrogenation [3,28]. The graphitization is the final step in the heat treatment. At this step, the ordering and orientation of the small turbostratic crystallites in the direction of the fiber axis take place [1,3]. From carbonization to graphitization, the size of the graphite layer structure presents an upward tendency [29].

The electrical resistance change method (ERCM) measures the state of the material or the environment through a change in the resistance of the carbon fiber. ERCM is widely used in carbon fiber reinforcing composites to monitor strain, failure and temperature. Forintos [30] made carbon fiber reinforcing composites as temperature sensors to measure the temperature during epoxy curing. The resistance of the carbon fiber declined approximately 2.5% when the temperature rose from 0 to 100 °C, which indicated that the error generated by the equipment had a great impact on the results. Therefore, to produce fibers that are more sensitive to temperature may be a better choice. It has been reported that when carbonized in the temperature range of 500 to 800 °C, the C content of fibers is less than 92 wt%, meaning that the fibers belong to the partially carbonized carbon fibers [31]. Pan et al. [32] discovered that partially carbonized carbon fibers showed negative temperature coefficients. Gillespie et al. [33] speculated that the electrical conduction in partially carbonized fibers was dominated by variable-range hopping in a temperature range of 2–300 K. This shows that the fibers carbonized in a temperature range of 500–800 °C are more suitable for making a temperature sensor.

In the paper, we explored the temperature sensitivity of different partially carbonized carbon fibers. Pre-oxidized fibers carbonized between 500 and 800 °C exhibited semiconductor characteristics. When the heat treatment temperature was less than 700 °C, the resistivity of the fibers was too great, which affected the test result due to self-heating during the test. Therefore, we chose 700, 750 and 800 °C as the heat treatment temperatures. In the actual carbonization process, the heat treatment temperature was usually short. Considering the reduction of energy consumption, we chose 3 and 9 min as the heat treatment temperature. The structures of the fibers were examined by infrared spectra, an element analysis, Raman spectroscopy, a SEM and X-ray diffraction. The electrical properties were characterized by measuring the resistivity of the samples.

## 2. Materials and Methods

### 2.1. Carbonization of the Pre-Oxidized Fibers

**Figure 1:**
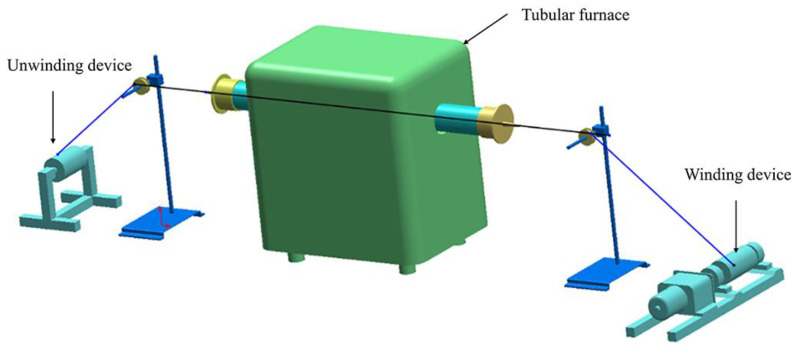
The equipment for preparing the carbon fiber.

Pre-oxidized fibers of grade 6 K (6000 filaments per tows) stabilized at 280 °C were provided by Jiangsu Hengshen Co. Ltd. (Jiangsu, China). The diameter of a single fiber was approximately 9 μm. The samples were carbonized by self-designed equipment that included a tubular furnace, a winding device and an unwinding device (Figure 1). The temperature of the tubular furnace could be adjusted from room temperature to 1000 °C. In the process, the fibers were heated under a nitrogen atmosphere. Six types of samples were prepared with different heat treatment temperatures. The heat treatment time is shown in Table 1.

### 2.2. Temperature Resistivity Measurements 

**Figure 2:**
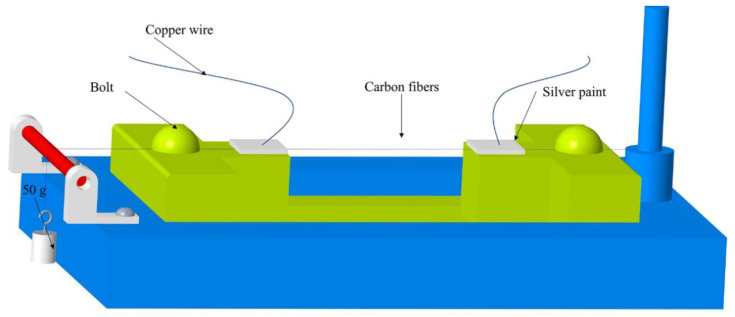
A scaffold for fiber testing.

The fibers, with a length of approximately 100 mm, were put on the test rack (shown in Figure 2) and a force of 490 N was applied to the end of the fibers to ensure the fiber had the same tension each time. To measure the resistivity of the fibers, silver paint was used to contact the copper wire and the fibers. The whole test rack was then put in the chamber at 105 °C for 2 h to fully cure the silver paint. After the paint was completely cured, two bolts were used to fix the fiber and the excess fibers were cut off.

The resistivity measurements of the samples were performed in a chamber. The temperature of the fibers was measured with a thermistor (PT-100) and a Keithley 2700 data acquisition module. The thermistor was attached to the middle of the rack to measure the temperature of the fibers. All specimens were measured in the same temperature range, which started at 25 °C as the initial temperature and finished at 100 °C. The data of temperature and resistivity were taken every 5 °C. In order to ensure the accuracy of the resistivity, the data were recorded when the change in temperature was less than 0.5 °C.

### 2.3. Characterization

Infrared spectra, element analysis, scanning electron microscope, Raman spectroscopy and X-ray diffraction measurements were used to characterize the chemical states, structure and composition of the samples.

## 3. Result and Discussion

### 3.1. Temperature Sensitivity of the Fibers

**Figure 3:**
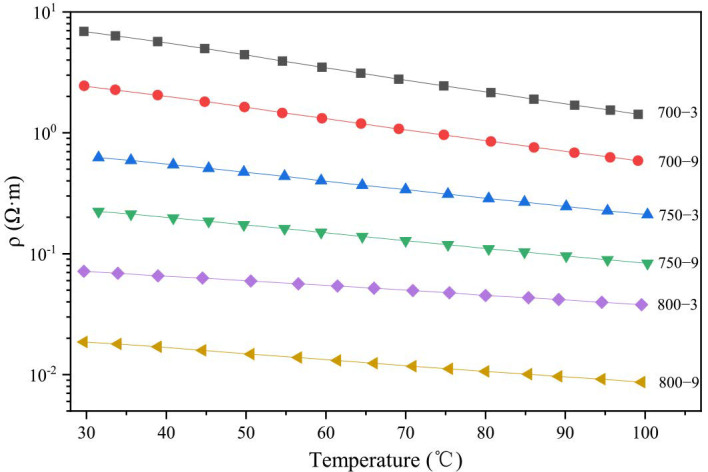
Temperature sensitivity of the samples.

The results, plotted in Figure 3, showed that the fibers were extremely sensitive to the heat treatment temperature and heat treatment time. The resistivity at 30 °C changed by almost 3 orders of magnitude with a 100 °C change in the heat treatment temperature. At the same heat treatment temperature, the resistivity of the samples changed almost 0.5 orders of magnitude with 6 min changes in the heat treatment time. This showed that the effect of the heat treatment time on the resistivity of the samples was much less than the heat treatment temperature.

As the test temperature increased, the resistivity of the fibers showed a downward trend. The samples showed negative temperature coefficients. Linear fits were performed on the data and all values of R^2^ were greater than 0.999. It indicated that there was a linear relationship between the temperature and the logarithm of resistivity. When the test temperature rose from 30 to 100 °C, the change in resistivity of samples 700-3, 700-9, 750-3, 750-9, 800-3 and 800-9 were approximately 79, 76, 66, 63, 53 and 47%, respectively, and the slope of the line gradually increased. From the above result, it could be seen that the specimens had a different temperature sensitivity and, as the heat treatment temperature rose, the temperature sensitivity of the samples gradually decreased.

### 3.2. The Effect of the Graphite-like Structure on the Temperature Sensitivity

**Figure 4:**
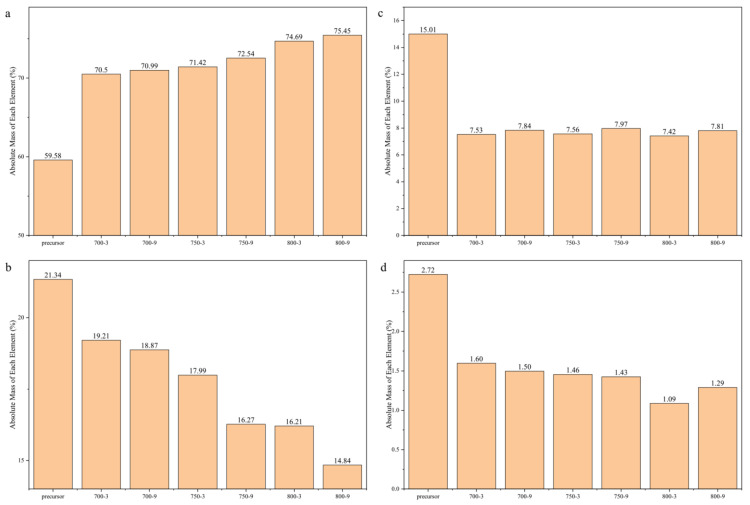
Element changes of the different carbon fibers: (**a**) C; (**b**) N; (**c**) O; (**d**) H.

**Figure 5:**
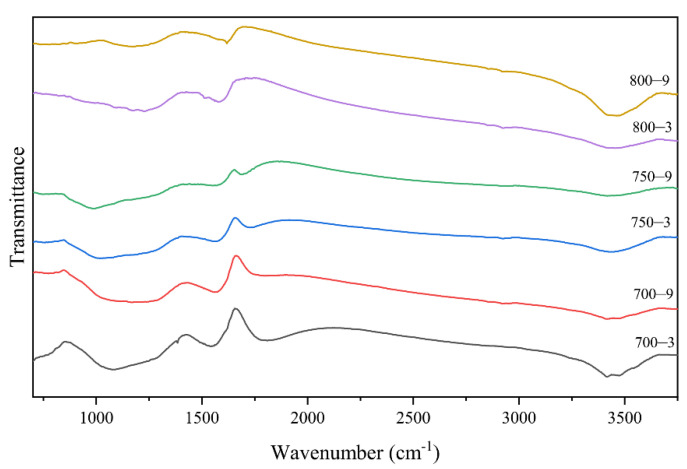
IR spectra of the samples.

Infrared spectra, a scanning electron microscope and an element analysis were used to characterized the proportion of graphite-like structures in the samples. In Figure 4a, it can be seen that the C content values of 700-3, 750-3, 800-3, 700-9, 750-9 and 800-9 were 70.5, 71.42, 74.69, 70.99, 72.54 and 75.45 wt%, respectively, which showed that the heat treatment temperature had a greater impact on the C content. As seen in Figure 4b,d, the N content of the samples showed a downward trend, which was attributed to the main reaction that released HCN. From Figure 5, it can be seen that with an increase in the heat treatment temperature and the heat treatment time, the absorption peak intensity of the organic functional groups gradually decreased and the intensity of the peak was around 1600 cm^−1^, attributed to C=N, N–H and C=N [34]. With a decrease, the peak moved to a higher wave number indicating that in the carbonization, the C content presented an upward trend. The N and H elements were reduced and the reaction of C=N and C–H produced graphitic-like structures followed by the formation of plain graphite layers. With a rise in the heat treatment temperature and the heat treatment time, the C content increased. This was attributed to the increase in the proportion of graphite-like structures. Combined with the data in Figure 3, it could be seen that as the proportion of graphite-like structures increased, the resistivity of the fibers decreased significantly. It could be inferred that the proportion of graphite-like structures could affect the temperature sensitivity of the samples.

As shown in Figure 4c, when the heat treatment time increased from 3 to 9 min, the O content of the fibers carbonized at the same temperature increase. When the heat treatment temperature increased from 700 to 800 °C, the O content of the fibers first increased and then decreased with the same heat treatment time. This was attributed to the main reaction, which occurred in the range of 700–800 °C and released HCN. The release reached a peak around 750 °C, which was the main reason for the variation of the H, O and N content. The intensities of the peak around 1700 cm^−1^ decreased, as shown in Figure 5, which was attributed to the C=O band [34]. This demonstrated that a few defects formed in the graphite-like structure in the process.

**Figure 6:**
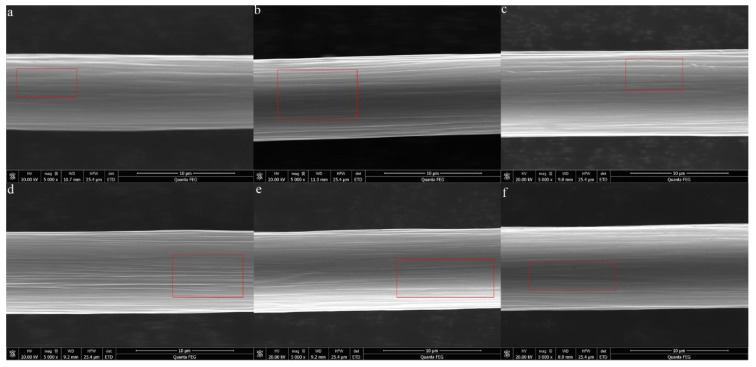
SEM images of the samples: (**a**) 700-3; (**b**) 700-9; (**c**) 750-3; (**d**) 750-9; (**e**) 800-3; (**f**) 800-9.

The SEM images of the samples are shown in Figure 6. These images showed that as the heat treatment temperature rose, the fold depth on the surface of the samples increased and the heat treatment time affected the number and width of the folds. With a rise in the time, the number and width of the folds increased. The folds were attributed to the main reaction, which indicated that gas was released on the surface of the specimens with an increase in the heat treatment temperature and time.

### 3.3. The Effect of the Graphitization Degree on the Temperature Sensitivity 

**Figure 7:**
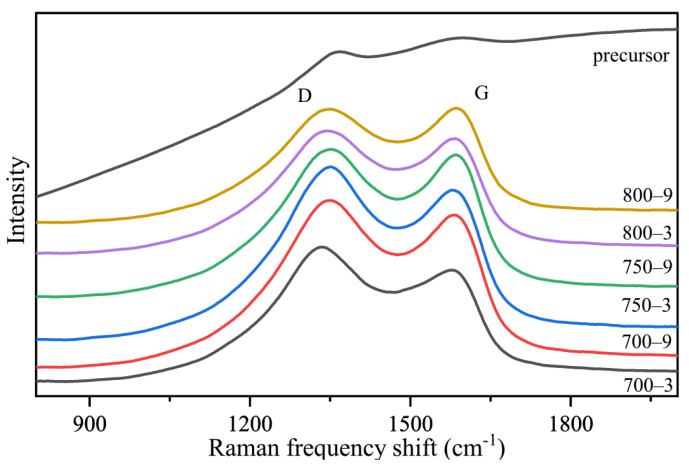
Raman spectra of the samples.

In Figure 7, it can be seen that there were two broad bands at 1360 cm^−1^ and 1580 cm^−1^. The G-line at 1580 cm^−1^ was produced by the graphite structure. The D-line appeared at 1360 cm^−1^, resulting from a low orientation, incomplete graphite crystallites and many structure defects in the samples. The intensity of the band (*I*_D_) was used to characterize the degree of the structural disorder. The degree of graphitization was characterized by the ratio of the integrated intensity of the D-line band to the G-line band (*R*). The greater the *R* value, the lower the graphitization degree. The formula is as follows [35]:

(1) $$R=\frac{I_{\;D}}{I_{\;G}}$$

In Figure 6, it can be seen that the *R* values of 700-3, 750-3, 800-3, 700-9, 750-9 and 800-9 were 1.16, 1.12, 1.05, 1.08, 1.03 and 0.99. This showed that an improvement in the heat treatment temperature or heat treatment time could increase the graphitization degree of the fibers and the heat treatment temperature had a greater impact on the graphitization degree. The full width at half maximum height (FWHM) of the G band and D represented the uniformity of the graphite structures and the disordered structures, respectively. As shown in Table 2, the FWHM (D band and C band) of 700-3, 750-3, 800-3, 700-9, 750-9 and 800-9 gradually decreased. This showed that the FWHM of the G band and D band showed a downward tendency as the heat treatment temperature and the heat treatment time rose, indicating that the increase in the heat treatment temperature or heat treatment time improved the structural homogenization of the fibers. 

**Figure 8:**
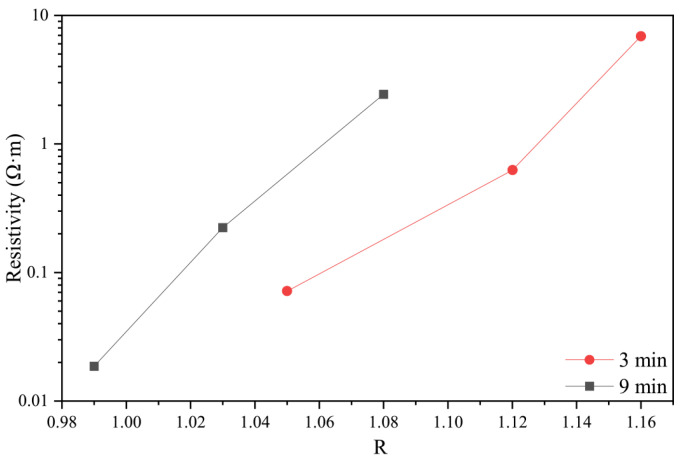
The effect of graphite degree on resistivity.

To explore the relationship between the graphitization degrees and the temperature sensitivity, the *R* value and resistivity at 30 °C were plotted, as shown in Figure 8. As the *R* value decreased, the resistivity of the specimens carbonized at the same time and showed a downward trend. There were also cases where the R value was small and the resistivity was large. Combined with Figure 3, it could be inferred that the temperature sensitivity could be affected not only by the degree of graphitization but also by other factors.

### 3.4. The Effect of the Graphite-like Crystallite Size on the Temperature Sensitivity

An X-ray diffractometer (Bruker D8 advance) operated at 40 kV and 150 mA with Cu *K_α_* radiation was used to measure the size of the graphite-like crystallite in the samples. The data were collected over a 2θ range of 5–90° at a scan rate of 5° min^−1^. The graphite crystallite interlayer spacing *d*_002_, crystallite width *L_a_* (*L_a_*_⊥_ and *L_a//_*) and crystallite thickness *L_c_* were calculated by the following equations [36,37]:

(2) $$d_{002}=\frac{\lambda }{2\sin\theta }$$

(3) $$L_{c}=\frac{K\;\lambda }{\beta \;\cos\theta }$$

**Figure 9:**
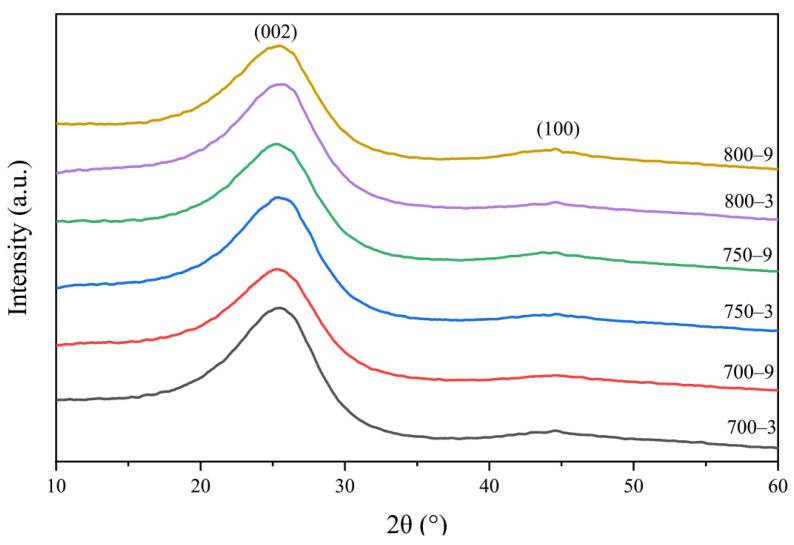
X-ray diffraction patterns of the samples.

In Figure 9, there are two peaks that appeared around 2θ = 25.5° and 44.5° in the XRD pattern, indicating that the lattice order was relatively low and could be regarded as graphite (002) and (100) planes [29]. The size of the graphitized-like crystallite was usually represented by *L*_c_, *L_a_*_⊥_, *L_a_*_//_ and *d*_002_.

The *L*_c_, *L_a_*_⊥_ and *L_a_*_//_ of the samples were calculated according to Equation (2) and the *d*_002_ was calculated according to Equation (3) [37]. In Table 3, the *L*_c_, *L_a_*_⊥_ and *L_a_*_//_ of 700-3, 750-3, 800-3, 700-9, 750-9 and 800-9 gradually increased and the *d*_002_ of 700-3, 750-3, 800-3, 700-9, 750-9 and 800-9 gradually decreased. The crystallite size of 700-3, 700-9, 750-3, 750-9, 800-3 and 800-9 gradually increased, indicating that the size of the graphite-like crystallite in the fibers gradually increased as the heat treatment temperature or the heat treatment time increased. In the experiment, the influence of the heat treatment temperature on the size of the crystallite was greater than the heat treatment time. Combining Figure 3 and Table 3, the change in the crystallite size was consistent with the change in resistivity of 700-3, 700-9, 750-3, 750-9, 800-3 and 800-9 when the test temperature increased from 30 to 100 °C. It indicated that the crystallite size may affect the temperature sensitivity in the proportion of the graphite-like structure.

### 3.5. Structure–Temperature Sensitivity Relationships

From the results, we inferred that the proportion of the graphite-like structure, graphitization degree and size of the graphite-like crystallite could influence the temperature sensitivity of the samples. The crystallite size had a greater influence on the temperature sensitivity. From a crystallite size analysis, we discovered that when the heat treatment temperature increased, the distance between the graphite layers decreased, the crystallite size increased and the graphitization degree of the overall structure increased. At the same time, a change in the resistivity of 700-3, 700-9, 750-3, 750-9, 800-3 and 800-9 decreased gradually when the test temperature increased from 30 to 100 °C, demonstrating that the crystallite size was the main structure that affected the formation of electron transport in the samples. 

**Figure 10:**
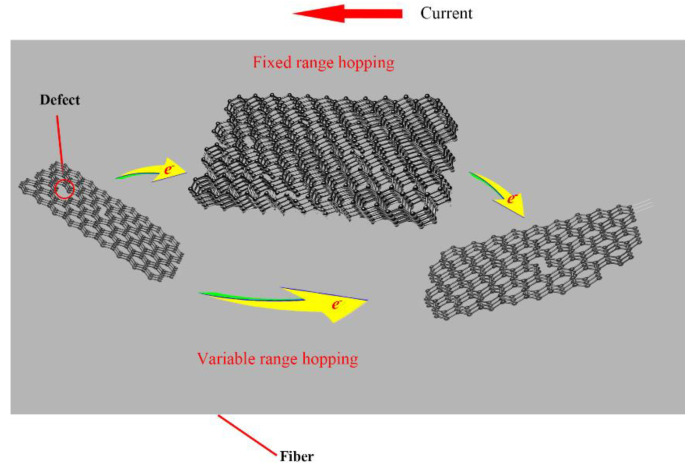
The electrical conduction mechanism in the samples.

When graphitized-like crystallite is heated, the σ bond breaks, making unpaired σ electrons appear around a ring aromatic structure. The π electrons will pair with the σ electrons to generate holes, which can migrate under the action of an electric field. The charge is transferred between the graphitized-like crystallite by hopping [38,39,40]. The electrical conduction resulting from such a hopping mechanism is shown in Figure 10. Fixed-range hopping (FRH) occurs at high temperatures and electrons mainly move between adjacent local states. Variable-range hopping (VRH) occurs at low temperatures and electrons can jump between localized states with relatively long distances and similar energies. The form for the conductivity from VRH is given by [33,41]:

*ρ* = *ρ*_0_exp(B/*T*)^1/4^ (4)

where B is a constant and *ρ*_0_ is a constant as the resistivity at an infinite temperature. The form for the conductivity from FRH is given by [32]:

*ρ* = *ρ*_0_exp(E/*kT*) (5)

where E and *k* are constants. 

**Figure 11:**
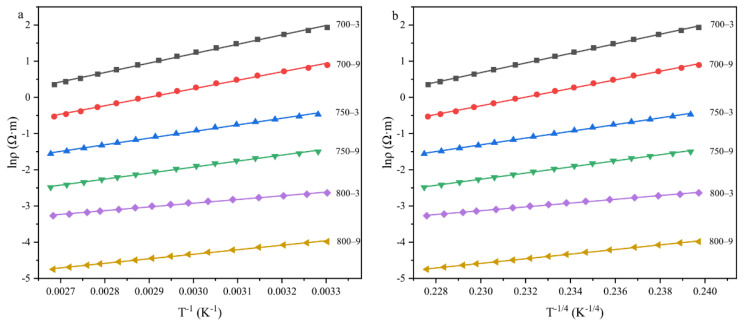
Temperature dependence of electrical resistivity: (**a**) fixed-range hopping used to linear fit; (**b**) variable-range hopping used to linear fit.

In order to explore the conduction mechanism of the fiber at room temperature, both VRH and FRH were used to linear fit. As shown in Figure 11a,b, we plotted the resistivity of the fibers as a function of *T*^−1^ and *T*^−1/4^. Through the analysis of the fitting residual and *R*^2^, the *R*^2^ value of FRH and VRH in the six groups was around 0.99 but the *R*^2^ value of VRH was larger than FRH and the residual value of VRH was smaller than FRH. This indicated that when the heat treatment temperature was in the range of 700–800 °C, the electronic transport of the samples at 30–100 °C contained FRH and VRH and VRH was the main result. Due to the two conductive mechanisms, the samples showed a temperature sensitivity.

## 4. Conclusions

The fibers, carbonized at 700–800 °C, showed temperature sensitivity. Through the analysis, the proportion of the graphite-like structure, graphitization degree and size of the graphite-like crystallite could influence the temperature sensitivity and the crystallite size had a greater influence. Combined with the structure, it could be inferred that the electron transport in the samples included FRH and VRH and VRH was dominant from linear fitting. This indicated that the increase in temperature promoted the electron transfer, making the fiber exhibit a temperature sensitivity.

## Figures and Tables

**Table 1:** The heat treatment temperatures and times of the fibers.

Sample ID	Heat Treatment Temperature (°C)	Heat Treatment Time (min)
700-3	700	3
700-9	700	9
750-3	750	3
750-9	750	9
800-3	800	3
800-9	800	9

**Table 2:** Raman spectra of the samples.

Sample	FWHM (D) (cm^−1^)	FWHM (G) (cm^−1^)	*R* (*I*_D_/*I*_G_)
700-3	282	375	1.16
700-9	271	344	1.08
750-3	277	359	1.12
750-9	270	309	1.03
800-3	273	348	1.05
800-9	265	299	0.99

**Table 3:** The *d*_002_, *L*_c_ and *L_a_* of the crystallite of the samples.

Sample ID	*d*_002_(nm)	*L*_c_ (002)(nm)	*L_a_*_//_(100)(nm)	*L_a_*_⊥_(100)(nm)
700-3	0.355	1.70	1.00	1.84
700-9	0.354	1.71	1.01	1.86
750-3	0.353	1.77	1.08	1.99
750-9	0.353	1.78	1.10	2.03
800-3	0.352	1.79	1.22	2.25
800-9	0.3251	1.81	1.24	2.28

## Data Availability

The evaluated data presented in this study are available in the tables of this paper. The raw measured data of this study are available on request from the corresponding author.
